# Comment on “Mitochondrial Neurogastrointestinal Encephalomyopathy: Novel Pathogenic Mutation in Thymidine Phosphorylase Gene in a Patient from Cape Verde Islands”

**DOI:** 10.1155/2021/5498640

**Published:** 2021-02-26

**Authors:** Josef Finsterer

**Affiliations:** Klinik Landstrasse, Messerli Institute, Vienna, Austria

With interest, I read the article by Falcão de Campos et al. about a 19-year-old Cape Verdean female with mitochondrial neurogastrointestinal encephalopathy (MNGIE) due to the variant c.1283G>A in *TYMP* [1]. The index patient was born to consanguineous parents and had a sister who was also clinically affected [1]. A number of additional points warrant consideration, as follows:

1. Mitochondrial DNA (mtDNA) was not investigated for point mutations or rearrangements. Since *TYMP* variants have been shown to secondarily cause mtDNA depletion or mtDNA deletions [2], it is crucial that investigations of the index patient's mtDNA were carried out.
2. Muscle biopsy was not investigated by electron microscopy. Even if histology and immune-histology are normal, ultrastructural abnormalities of mitochondria regarding shape and structure (paracrystalline, mitochondrial inclusions, abnormally shaped mitochondria, parking lots, distorted cristae formation, lipid or glycogen droplets) may be seen. Since the patient had bilateral ptosis, quadriparesis, a myopathic needle electromyography, and lactic acidosis, it is conceivable that ultrastructure of myocytes was abnormal.
3. No biochemical investigations of the muscle homogenate had been carried out. Biochemical investigations are crucial to see which of the respiratory chain complexes are dysfunctional and to which degree their activity is reduced.
4. Parents and other first-degree family members, in particular, the clinically affected sister, were neither clinically nor genetically investigated. Knowing the genetic status of first-degree relatives is crucial for genetic counselling, to assess the phenotypic variability, to determine the disease course, to assess the pathogenicity of the variant, and to predict the outcome of the individual patient.
5. The authors did not clarify if gastrointestinal compromise was due to affection of the smooth muscle cells or due to affection of the autonomic nervous system.
6. The activity of TYMP and serum levels of the nucleosides dThd and dUrd were not determined. Knowing the enzyme activity and the nucleoside levels is crucial for assessing any therapeutic effect, as they may serve as biomarkers of the disease.
7. The index patient and her sister did not receive any of the four established therapies for MNGIE [3]. These include hemodialysis, liver transplantation, autologous hematopoietic stem cell transplantation, or erythrocyte encapsulated TYMP. It would be of interest to know why no therapy was established.
8. The cause of death was not mentioned, and no autopsy investigations were carried out. Since MNGIE patients may also manifest in the heart [4], it is crucial to know if there were any indications for clinically manifesting or subclinical cardiac compromise. It should be discussed if ventricular arrhythmias, cardiac arrest, or cardiomyopathy were present and could be considered responsible for the early death of the patient.

In summary, before reaching a final conclusion, the above points should be more completely addressed. Since MNGIE patients are rare, there is an urgent need to thoroughly investigate these patients intra vitam and post mortem for genetic and clinical peculiarities. Additional information about secondary mtDNA depletion or deletions, ultrastructural changes, biochemical investigations, first-degree relatives, the cause of gastrointestinal involvement, the index patient's parents and sister, why MNGIE was not therapeutically treated, and whether there was any cardiac involvement is desirable. This information would be very helpful in understanding the disease process and in assessing the outcome.
